# More Than Just a Room: A Scoping Review of the Impact of Homesharing for Older Adults

**DOI:** 10.1093/geroni/igaa011

**Published:** 2020-05-03

**Authors:** Laura Martinez, Raza M Mirza, Andrea Austen, Jessica Hsieh, Christopher A Klinger, Michelle Kuah, Anna Liu, Lynn McDonald, Rida Mohsin, Celeste Pang, Jennifer Rajewski, Tonya Salomons, Iqra Sheikh

**Affiliations:** 1 Factor-Inwentash Faculty of Social Work, University of Toronto, Canada; 2 National Initiative for the Care of the Elderly (NICE), Toronto, Canada; 3 Seniors Transition Office, City of Toronto, Canada; 4 Department of English, University of Toronto, Canada; 5 Department of Anthropology, University of Toronto, Canada

**Keywords:** Aging in place, Housing, Intergenerational

## Abstract

**Background and Objectives:**

“Aging in place” is commonly defined as the ability to remain living safely and independently for as long as possible either in the home or community of one’s choosing. Yet, the literature indicates that older adults prefer to remain specifically in their own homes. Homesharing, an innovative exchange-based housing approach, is a means by which older adults can obtain additional income, companionship, and assistance by renting out a room to a home seeker, potentially increasing capacity to remain living independently in their homes. But what is known about their experiences of homesharing?

**Research Design and Methods:**

A scoping review was conducted to map and consolidate the literature related to the experience of homeshare participation for adults aged 55 and older published from 1989 to 2018. Fifteen databases were searched, including 3 medical, 5 social science, and 7 gray literature databases. Following abstract and full-text review, 6 sources were retained for study inclusion. Thematic content analysis was used to identify major themes.

**Results:**

Within included studies, 4 major themes were identified: (i) benefits of homeshare participation for older adults; (ii) challenges of participating in homeshare for older adults; (iii) intergenerational engagement as social exchange; and (iv) the key role of agency facilitation.

**Discussion and Implications:**

Findings were used to derive practice, policy, and research implications. By focusing on older adults and the ways homesharing impacts their lives, we can better determine the viability of homeshare as a means for improving and prolonging experiences of living at home.

**Translational Significance:** Homesharing is an innovative, often intergenerational, exchange-based housing model in which a home provider, often an older adult, shares a spare room in their home with a home seeker in exchange for money, service provision, or a combination of the two. Results indicated that older adults benefitted from the increased companionship, support in completing daily tasks, and increases in well-being associated with homesharing. Navigating boundaries with respect to sharing space, time, and interpersonal relationships was a challenge when homesharing. Agency facilitation was reported as key to supporting a positive homeshare experience for older adults. Policy implications include the potential for homesharing to save health and social services spending.

The home carries particular psychosocial and material significance in later life. Psychological attachment to home increases with age and length of time in residence, with many older adults feeling strongly about staying in their home. Research by Després and Lord (2005) on the meaning and experience of home found that older adult respondents viewed the home as a locus of physical and psychological security, an indicator of social status, a venue for social engagement, a place where mobility is practiced, a central hub of day-to-day life, and a site of familiarity, attachment, and memory. Experiencing *home* may be integral to family tradition, where the physical space of the home represents an accumulation of special moments and memories (O’Bryant & Wolf, 1983). Additionally, the home can represent a major financial resource for many older adults due to the proportionally high rate of homeownership and low rate of mortgage debt among this population (Danigelis & Fengler, 1990). According to Statistics Canada (2017b), 76.3% of individuals aged 55–64 own their home. Among households with a homeowner aged 60–69, 74% were mortgage-free; among households with an owner aged 70 and older, roughly 90% are mortgage-free (Brown, Hou, & Lafrance, 2010). However, housing may become unsafe and financially burdensome for property-rich, cash-poor older adults who struggle with the maintenance of their homes and the costs associated with repairs and upkeep, accessibility barriers with respect to mobility within their home, or social isolation or loneliness (Employment and Social Development Canada, 2019; National Seniors Council, 2014).

## Aging in Place

In a recent survey conducted by the Canada Mortgage and Housing Corporation (CMHC, 2008), more than 85% of those aged 55 and older reported that they intended to continue living in their current home, or “age in place” for as long as possible, irrespective of changes in their health. A much-cited 2014 AARP survey found that 88% of respondents over the age of 65 in the United States endorsed a desire to remain in their residence for as long as possible (Barrett, 2014, p.4). Similarly, a 2015 survey from the United Kingdom indicated that 80% of older homeowners prefer to remain in their current home (Lloyd & Parry, 2015).

As part of a growing movement and as it is understood, aging in place highlights “having the health and social supports and services you need to live safely and independently in your home or your community for as long as you wish and are able” (Social Development Canada, 2016, p. 2). Although the concept of aging in place should be understood as encompassing both the home and its surrounding dwelling and community (Wiles, Leibing, Guberman, Reeve, & Allen, 2012), studies showed that older adults prefer to remain in their current homes specifically (Bayer & Harper, 2000; CMHC, 2008).

Aging in place allows older adults to remain independent, autonomous, and connected to their social network (Callahan, 1993; Keeling, 1999). However, as individuals experience losses related to the aging process, such as a decline in mobility, health status, income, and available social supports, the capacity to live safely and independently becomes increasingly limited (Danigelis & Fengler, 1990). When environmental demands exceed an individual’s capacity to meet them, they are said to experience “environmental pressures” (Lawton, 1980, p. 13). Without access to appropriate supports and services to help meet their increased needs and relieve environmental pressure, such as assistance with meal preparation, housekeeping, or with shopping and errands, older adults were left with few options and may face inappropriate institutionalization (Rekart & Trevelyan, 1990). In this sense, aging in place presents the challenge of how to ensure that older adults are not merely *staying* in their homes, but rather, are able to remain safe, autonomous, and socially engaged.

## Homeshare: An Alternative Living Arrangement Supporting Aging in Place

While the preference of older adults is to remain in their homes (CMHC, 2008) when their ability to remain there becomes compromised, other options must be made available. As there are fiscal and ethical concerns due to the high cost of long-term care and the risk of premature institutionalization for older populations (Rekart & Trevelyan, 1990), various alternative housing initiatives have been developed to prolong independence and to support aging in place. One such initiative is homesharing: “a living arrangement in which unrelated people, not necessarily seniors, occupy a single dwelling, sharing common areas such as a kitchen, bathroom, and living room, but each maintaining a private space as well” (Gutman, Doyle, Melliship, & Baldwin, 1989).

Whereas boarding arrangements of this nature have existed throughout history, the use of formal programs to arrange homesharing matches as a means of providing older adults with the necessary income, companionship, and assistance with activities of living independently necessary for them to remain in their home has only existed in Canada since 1980 (Johnstone, 2001), and in the United States since the 1970s (Gutman et al., 1989). According to HomeShare International (n.d.), the first homesharing program was adopted in 1991 in Spain, 1992 in Germany, and 1993 in the United Kingdom and, as of April 2017, there are formal homesharing programs in 16 countries across the globe, including Australia, North America (Canada, United States), across Europe (Austria, Belgium, France, Germany, Republic of Ireland, Italy, Spain, Switzerland, and the United Kingdom), and in Asia (Korea and Japan), commonly involving the matching of older home providers with younger home seekers.

Beyond the simple sharing of space, homesharing is transactional, operating on principles of social exchange (Danigelis & Fengler, 1990) between the matched participants (“homesharers”), recognizing that each participant has both needs *and* something to offer. An exchange agreement between homesharers outlines the conditions of the homeshare arrangement, usually involving a person agreeing to provide shelter (“home provider”) to a person seeking to move into the home (“home seeker”) in exchange for money, service provision, or a combination of the two (Gutman et al., 1989). Research indicates that older adults are primarily motivated to participate in homeshare for companionship, financial assistance, help with household tasks, and increased sense of security (Danigelis & Fengler, 1991; Gutman et al., 1989; Howe, 1985; Jaffe, 1989a).

The literature commonly refers to Schreter’s (1986) typology of homeshare: (i) self-initiated or naturally occurring, involving homesharing agreements initiated privately by participants without third-party involvement; (ii) agency-sponsored, whereby organizations purchase or build a home and recruit participants to cohabitate there; and (iii) agency-assisted homeshare, where an organization, often nonprofit, facilitates the homesharing process (Gutman et al., 1989; Johnstone, 2001). However, the level of support and oversight on the part of the agency can vary considerably: in some cases, agency facilitation may be limited to maintaining a housing registry and providing contact information to prospective homesharers, while tasking the homesharers themselves with creating and maintaining a match (referred to as a “referral model”); in other cases, dedicated homeshare program staff will provide extensive facilitation (hereupon referred to as the “facilitated model”), often including prescreening and matching participants, follow-up, mediation, and termination services (Gutman et al., 1989).

Homeshare is simply one option of many communal living options. Resident-managed elder intentional neighborhoods, co-housing, Elder Cottage Housing Opportunities, and “granny flats” are shared housing arrangements with many of the same aims and potential benefits of homeshare: prolonging independence, increasing social engagement, supporting autonomy, and ultimately enabling older adults to remain in the community (Jaffe & Howe, 1988). However, with the exception of the agency-sponsored model, homesharing does not necessitate the building of new infrastructure, housing developments, or additions to the home, making homesharing the least costly alternative. Most importantly, homesharing is unique among other community-based shared housing options insofar as it does not require a participant to move out of their home, therefore supporting their preferences and right to autonomy (Jaffe & Howe, 1988). Homeshare is singular in its potential to empower older adults to obtain the social supports and supplemental income they need to increase their capacity to cope with the demands of their environment—to live where they want to live—by creating a means for them to leverage a resource they already have: their home (Danigelis & Fengler, 1990).

With the exception of research by Danigelis and Fengler (1990, 1991), Jaffe (1989a, 1989b), and Sánchez, García, Díaz, and Duaigües (2011), evaluations of homeshare programs in relation to their impacts on the lives of older adults are scarce. However, existing research suggests that older participants are broadly satisfied with their homeshare experiences (Howe, 1985; Rekart & Trevelyan, 1990). Surveys conducted by Altus and Mathews (2000) found that more than half of home providers aged 50 and older reported feeling safer, happier, and less lonely as a result of participating in intergenerational homeshare. However, much of the literature on homeshare is process-oriented, describing homeshare program design and implementation, best practices, and client demographic information (e.g., Gutman et al., 1989; Pritchard & Perkocha, 1989; Robins & Howe, 1989). Although this work is inarguably necessary and valuable, there is little research examining the specific ways in which these programs impact the lives of those for whom they were designed to benefit.

In order to identify and address gaps in knowledge, a comprehensive scoping review was undertaken to explore the current state of evidence and research on the impacts associated with participation in homeshare for older adult populations. This review seeks to describe and synthesize the existing literature in this area and to identify current best practices and interventions. Taken together, findings were used to derive implications for practice, policy, and areas for future inquiry.

## Method

A scoping review methodology was chosen because its framework serves to map the literature, to examine and summarize the nature of research being conducted, to disseminate findings, and to identify gaps in the existing literature (Arksey & O’Malley, 2005), and to position the knowledge available within a context of research, policy, and practice implications (Arksey & O’Malley, 2005; Levac, Colquhoun, & O’Brien, 2010). This review was conducted following the six-stage framework for scoping reviews as outlined by Levac and colleagues (2010): (i) identifying the research question; (ii) identifying relevant studies; (iii) study selection; (iv) charting the data; (v) collating, summarizing, and reporting results; and (vi) expert consultation. The research question of our scoping review was: *“What is known about the impacts associated with participation in homesharing for older adults aged 55 and older?”* The following subquestions were also identified: (i) What is known about the benefits and challenges of engaging in homeshare for an older adult population? (ii) What is known about the factors supporting or inhibiting positive experiences of homesharing for older adults? (iii) What are the implications for policy and practice? (iv) What are the gaps in existing research and implications for future research?

### Eligibility Criteria

An important methodological consideration in this review was the range of possible models of homeshare (Gutman et al., 1989), as well as considerable conceptual overlap between formal homesharing initiatives, informal shared living arrangements (such as an older adult living with a family member), and other community-based shared housing arrangements in terms of being predicated on the sharing of space between unrelated participants as per the definition by Gutman and colleagues (1989), an element of exchange or mutual support, and with the aim of prolonging independence. As such, several search terms related to homeshare and shared housing were included in the search strategy to capture the literature on homesharing in accordance with our working definition (Table 1). Additionally, in order to capture all available findings related to outcomes of homesharing, we did not delineate or search for specific outcomes or impacts of homesharing within our search strategy, as a preliminary search revealed that this narrowed results considerably and would have excluded unanticipated outcomes and impacts. The search terms and subject headings used for the search strategy were identified by conducting a preliminary literature review and finalized through expert consultation from a University of Toronto librarian. Sources were eligible for inclusion if the concept of homeshare was (i) addressed as per the definition by Gutman and colleagues (1989), (ii) homeshare home providers were at least 55 years of age and older, and (iii) an evidence-based impact of participating in homeshare for our target population was discussed. Exclusion criteria consisted of (i) non-English language sources, (ii) sources whereby the participants did not meet our age criteria, (iii) sources pertaining to housing arrangements that did not fit our working definition of homeshare as per Gutman and colleagues (1989 p. 1), (iv) sources that did not include an evidence-based impact of homesharing, and sources published prior to 1989, as fundamental works by Gutman and colleagues (1989) synthesized the relevant literature prior to that year.

**Table 1.** Search terms for literature on homeshare and older adults

Conceptual term	Specific terms
*Homeshare*	(home* shar* OR homeshar* OR home-shar* OR shar* home* OR sharehome OR flat shar* OR shar* flat OR flat-shar* OR share* hous* OR hous* shar* OR house-shar* OR houseshar* OR communal hous* OR communal living OR roommate* OR room mate* OR room-mate* OR housemate* OR house mate* OR house-mate* OR cohousing OR co-housing)
*Population*	AND (older adult* OR older people OR older person OR senior* OR elder* OR aging OR geriatric OR gerontolog*)

### Search Strategy

A computerized search of the literature was conducted between October and December 2018. A total of 15 electronic databases were searched, including eight major academic medical and social sciences databases: AgeLine, Applied Social Sciences Index and Abstracts (ASSIA), CINAHL, Medline, PsycINFO, Social Services Abstracts, Social Work Abstracts, and Sociological Abstracts. Seven gray literature databases were searched for governmental and institutional publications, program evaluations, and unpublished academic work and dissertations. Keyword syntax was adapted according to the search parameters of each database (see Supplementary Appendix B for additional details). Finally, reference lists from selected sources and publications of relevant stakeholder organizations were searched manually to identify and curate relevant sources that may not have been located through our database searches. An expert consultation was conducted in December 2018 and served to validate search strategy results.

### Study Selection

**Figure 1.**
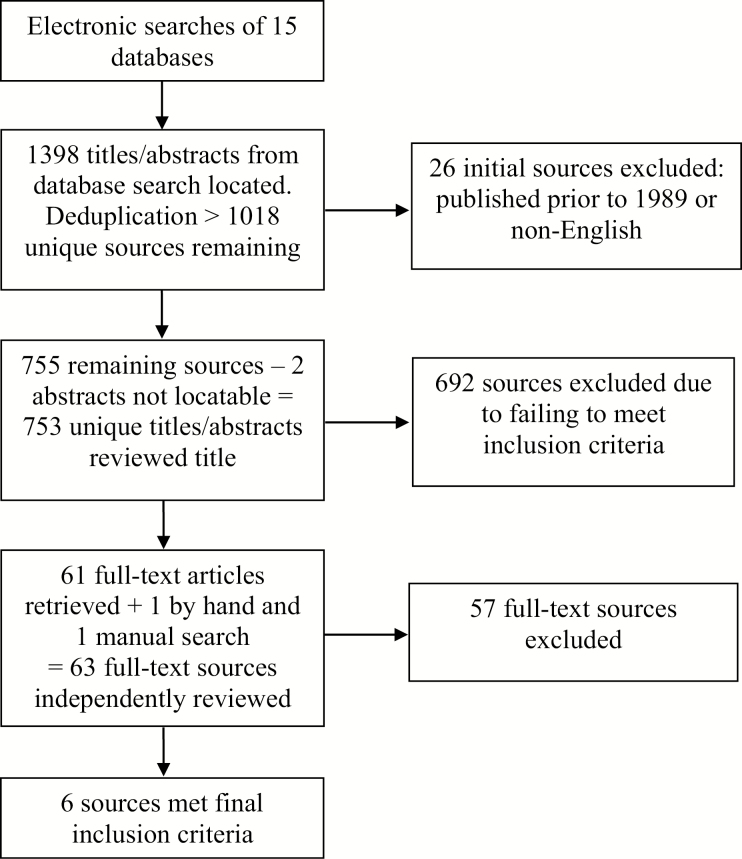
Flow chart of the study selection process.

In total, 1,398 sources, including articles, books, reports, and dissertations were located by the search strategy (Figure 1). After removing duplicates, 1,018 unique sources were retrieved.

All titles and abstracts generated by the search strategy were independently evaluated for relevance and adherence to exclusion and inclusion criteria by five reviewers (I. Sheikh, J. Rajewski, M. Kuah, R. M. Mirza, and T. Salomons). Full-text copies of sources with titles and abstracts fulfilling initial inclusion criteria were obtained for review. Final selection was then determined through full-text review and on the basis of adherence to inclusion and exclusion criteria. All discrepancies regarding study relevance and final selection were resolved by one reviewer (L. McDonald) in order to ensure consistency. A total of 263 sources were eliminated as per the exclusion criteria. Of the remaining 755 sources, abstracts for two titles (Dalley, Peretz-Brown, & Seal, 1995; Thornton, 1995) could not be located, and repeated efforts to locate the abstracts or full text of these reports, including contacting the relevant publishers, were unsuccessful. Screening of titles and abstracts of the remaining 753 sources resulted in 692 being excluded due to failing to meet the inclusion criteria, predominantly in terms of pertaining to shared housing arrangements that do not meet our definition of homeshare (i.e., co-housing) and failing to include an evidence-based outcome as a result of engaging in homesharing (i.e., simply explaining the concept of homesharing). The remaining 61 sources underwent full-text review. An additional source (Rekart & Trevelyan, 1990) was found by hand during this process and also reviewed in full. A total of 57 sources failed to meet inclusion criteria and were excluded following full-text review. A manual search of reference lists among the remaining five sources yielded two articles, which were subsequently reviewed in full. Of these two, one met inclusion criteria. A final total of six sources were retained for study inclusion.

Figure 1 illustrates the inclusion and exclusion assessment process of the sources located by the search strategy.

### Data Extraction

Important information from each selected source was organized and charted as per the categories proposed by Arksey and O’Malley (2005). Identified key themes were extracted and charted for the purpose of a thematic content analysis (Braun & Clarke, 2006).

## Results

After a thorough assessment of all available studies, six sources were included in the scoping review (Altus & Mathews, 2000; Bodkin & Saxena, 2017; Labit & Dubost, 2016; Macmillan, Gallagher, Ronca, Bidey, & Rembiszewski, 2018; Rekart & Trevelyan, 1990; Sánchez et al., 2011). Of the included sources, two were found through CINAHL (Labit & Dubost, 2016; Sánchez et al., 2011), two by manual searches (Altus & Mathews, 2000; Rekart & Trevelyan, 1990), and the rest were located through Ageline (Bodkin & Saxena, 2017) and Google Advanced Scholar (Macmillan et al., 2018) (see Supplementary Appendix A for characteristics of final selected sources). The majority were peer-reviewed (Altus & Mathews, 2000; Bodkin & Saxena, 2017; Labit & Dubost, 2016; Sánchez et al., 2011). The gray literature was comprised of reports evaluating homeshare agencies (Macmillan et al., 2018; Rekart & Trevelyan, 1990). Geographically, two of the sources originated from the United States (Altus & Mathews, 2000; Bodkin & Saxena, 2017), and the rest were from Canada (Rekart & Trevelyan, 1990), France (Labit & Dubost, 2016), Spain (Sánchez et al., 2011), and the United Kingdom (Macmillan et al., 2018).

Regarding research methodologies, two studies utilized a cross-sectional survey design (Altus & Mathews, 2000; Sánchez et al., 2011), two studies used qualitative designs consisting of face-to-face interviews (Bodkin & Saxena, 2017; Labit & Dubost, 2016), and two employed a mixed-methods approach involving analysis of agency data (Macmillan et al., 2018; Rekart & Trevelyan, 1990) combined with either qualitative interviews (Macmillan et al., 2018) or questionnaires (Rekart & Trevelyan, 1990). All sources studied agency-assisted homeshare, and all but one explicitly specified falling under the facilitated model of homeshare (Altus & Mathews, 2000; Bodkin & Saxena, 2017; Labit & Dubost, 2016; Macmillan et al., 2018; Rekart & Trevelyan, 1990). See Supplementary Appendix A for an outline of the characteristics and key findings of the included sources.

The results presented in this section are a summative content analysis of the final curated sources. The following four major themes were identified with respect to the research question of our scoping review: (i) benefits of homeshare participation for older adults; (ii) challenges of participating in homeshare for older adults; (iii) intergenerational engagement as social exchange; and (iv) the key role of agency facilitation as a determinant of the experience of homesharing for older adults.

### Benefits of Homeshare Participation for Older Adults

All included studies found that elder home providers explicitly reported benefitting from their participation in homesharing (Altus & Mathews, 2000; Bodkin & Saxena, 2017; Labit & Dubost, 2016; Macmillan et al., 2018; Rekart & Trevelyan, 1990; Sánchez et al., 2011).

#### Benefits associated with companionship

Results by Macmillan and colleagues (2018) identified “simply having someone to talk to on a regular basis” as a key benefit for many of the interviewed home providers, who described this increased engagement as reducing previous feelings of loneliness and social isolation (p. 26). Findings comparing baseline and endline home seeker and home provider UCLA loneliness scale measures suggested a reduction in the perception of loneliness as an outcome of participating in the homeshare program (Macmillan et al., 2018). Two studies reported a variety of positive impacts of homesharing as falling under the broad category of well-being: the majority (59.5%) of home providers aged 55 and older expressed an increased sense of well-being resulting from “companionship/reduced loneliness/better quality of life” (Rekart & Trevelyan, 1990, p. 43), and several home providers cited experiencing improved well-being in terms of improved sleep, reduced anxiety, increased motivation to engage in activities, and leaving the house more often (pp. 24–25). Home providers aged 70 and older were significantly more likely to report positive changes in domains of well-being (including feeling safer and liking living in their homes more), health (eating better), and social activities (following the news more closely) as a result of homesharing when compared with home providers aged 50–69 (Altus & Mathews, 2000). However, it should be noted that Altus and Mathews (2000) also found that, within the social response dimension, this same older group of home providers were more likely to report a negative change with respect to watching more television as a result of homesharing when compared with the younger cohort of home providers. Further benefits of homeshare reported by home providers include an increased sense of safety resulting from the reassuring presence of having another person in the home (Labit & Dubost, 2016) and security (Rekart & Trevelyan, 1990) as reported by 29.7% of respondents.

#### Benefits associated with receiving support in daily tasks

Findings indicated that help with personal care activities (Macmillan et al., 2018; Sánchez et al., 2011) and housekeeping tasks (Macmillan et al., 2018; Rekart & Trevelyan, 1990) represented important services for older adult home providers. The majority of home providers spoke of the value in receiving help with tasks of daily living, such as cleaning, cooking, shopping, and gardening, as well as with housekeeping activities for which home providers expressed functional limitations due to physical mobility issues, such as carrying shopping bags. Findings from three studies spoke to home providers aged 55 and older benefiting from enhanced independence (Labit & Dubost, 2016; Rekart & Trevelyan, 1990; Sánchez et al. 2011). Rekart and Trevelyan (1990) found that 22.6% of matched home providers mentioned enhanced independence as an advantage of their experience with homesharing.

In examining the impact of homesharing for clients of a Spanish homeshare program “Viure i Coniviure” (ViC), Sánchez and colleagues (2011) found that elder ViC participants reported greater capacity to accomplish tasks of daily living, such as attending a doctor’s appointment (35.1%), shopping (28.9%), completing household tasks (25.7%), running errands (25.7%), leaving the house (24.2%), and preparing meals (10.8%), in comparison with Spanish older adults of the same age and that these results persisted even when the comparison sample was controlled for marital status, concluding that the increased capacity of the ViC elders was associated to services provided by the home seekers in their home.

#### Financial benefit of homesharing

Bodkin and Saxena (2017) found that financial incentive—in the form of help paying for utilities and the relative inexpensiveness of receiving support through a homeshare exchange agreement relative to the cost of hiring private help—was a motivator for homesharing. However, results from three other studies suggest a negative correlation between self-reported importance of financial incentive and age (Altus & Mathews, 2000; Macmillan et al., 2018; Rekart & Trevelyan, 1990), whereas the older the homeowner was, the less important the financial aspect of the exchange arrangement or reported benefits from financial aspect of exchange. While Rekart and Trevelyan (1990) found that reduced cost of housing was the benefit most often (55.6%) expressed by matched home seekers, the majority of whom were under the age of 55 (92.6%), only 5.4% of home providers self-reported a financial benefit. Similarly, in endline interviews from their program evaluation, Macmillan and colleagues (2018) noted low cost of accommodations as a key self-reported benefit of participating in homesharing for home seekers, adding that home providers were aware that this was an important benefit for their home seekers, but no mention was made of home providers viewing the supplemental income they received as a benefit for themselves. Furthermore, Altus and Mathews (2000) found that, compared with respondents aged 50–69, home providers aged 70 and older were significantly less likely to report “worrying less about money” and “being better off financially” as a result of taking part in homesharing (p. 143).

### Challenges of Participating in Homeshare for Older Adults

Four studies cited self-reported disadvantages (Rekart & Trevelyan, 1990) or challenges (Bodkin & Saxena, 2017; Labit & Dubost, 2016; Macmillan et al., 2018) associated with homesharing for home providers aged 55 and older.

#### Navigating boundaries: Sharing space and time

Three studies spoke to challenges experienced with sharing space and coordinating time for support as expressed by elder home providers in terms of loss of privacy and control over the household (Bodkin & Saxena, 2017; Labit & Dubost, 2016; Rekart & Trevelyan, 1990), difficulty adjusting to the presence of another person in the home (Macmillan et al., 2018; Rekart & Trevelyan; 1990), lifestyle incompatibility (Bodkin & Saxena, 2017; Labit & Dubost, 2016; Rekart & Trevelyan, 1990), and conflicting expectations on shared time (Bodkin & Saxena, 2017; Macmillan et al., 2018).

With regards to sharing space, loss of privacy and control over the household was the category most often expressed (32.4%) by matched home providers when asked about disadvantages to homesharing (Rekart & Trevelyan, 1990). Elsewhere, qualitative interviews indicated that loss of household control was (i) expressed as a fear of potential disturbances or unwanted visitors (Labit & Dubost, 2016), and (ii) experienced as frustration over perceived lack of cleanliness on the part of the home seeker (Bodkin & Saxena, 2017). Similarly, adjusting to another person was a challenge: (i) expressed by 8.1% of matched home providers (Rekart & Trevelyan, 1990); and (ii) for widowed home providers especially at the beginning of the living arrangement (Macmillan et al., 2018). Incompatibility with respect to lifestyle was a challenge in two studies, cited as (i) a reason for match termination by one respondent (10%) due to the home provider’s discomfort with the home seeker’s alcohol consumption (Bodkin & Saxena, 2017); and (ii) as a disadvantage to homesharing by 5.4% of matched home providers (Rekart & Trevelyan, 1990).

Sharing time also emerged as a key issue in homeshare arrangements. Whereas Rekart and Trevelyan (1990) found that 61% of home providers expressed satisfaction with companionship and reduction of loneliness, two studies cited home provider dissatisfaction with companionship received (Bodkin & Saxena, 2017; Macmillan et al., 2018). Home providers expressed that the home seeker’s schedule (92% of all home seekers worked full time) often meant that they were left alone and without help during weekdays, and home providers gave several examples of home seekers feeling too tired to engage in activities with them after work or on their days off (Macmillan et al., 2018). Results from two studies spoke to home provider needs exceeding levels of support that could be provided within the context of the homeshare exchange arrangement: 43% of home seekers felt obligated to provide services beyond their agreed-upon time commitment of 10 hours, most often occurring within matches where the home provider had an existing higher level of need or where the home provider’s needs increased over the course of the match (Macmillan et al., 2018). In research by Bodkin and Saxena (2017), both home providers who were blind reported experiencing loneliness, boredom, and an increased need for human interaction, with one of these respondents having elected to leave the homeshare program and hire his former home seeker as a live-in companion (Bodkin & Saxena, 2017).

#### Navigating interpersonal boundaries

Results from two studies spoke to intermatch difficulties with respect to interpersonal relations and communication (Bodkin & Saxena, 2017; Macmillan et al., 2018).

#### Discomfort with the unfamiliar

In research by Bodkin and Saxena (2017), 3 of 10 home providers cited challenges with respect to the mental health of their matched home seeker. These home providers reported hosting home seekers whom they perceived as experiencing mental health difficulties. Each home provider experienced having a home seeker with perceived mental health challenges differently, depending on their own level of experience and familiarity with engaging with someone with mental health issues, as well as their level of comfort seeking match support. Out of these home providers, one remained living harmoniously with her home seeker due to her own reported experience caring for someone with mental illness; one home provider reported feeling considerable discomfort with her home seeker’s perceived hoarding behavior but did not seek a rematch, electing instead to avoid confrontation and remain living with her homeseeker despite considerable tension; the third home provider contacted agency staff when she felt she could not accommodate what she perceived to be the home seeker’s mental health challenges and she was re-matched and subsequently satisfied with the results. Furthermore, one home provider reported feeling uncomfortable living with her transgender homeseeker, describing her “‘lack of familiarity’ with individuals who are transgender” as a “‘barrier’ in their relationship” (Bodkin & Saxena, 2017, p. 52). However, despite her reported discomfort, this home provider was also not comfortable notifying agency staff to be re-matched with a new homesharer.

#### Challenges in communication

Two studies cited difficulties with intramatch communication (Bodkin & Saxena, 2017; Macmillan et al., 2018). Several home providers reported feeling awkwardness in relation to directly addressing issues within the homesharing process, included asking home seekers to complete agreed-upon household tasks and enforcing house rules (Macmillan et al., 2018). Bodkin and Saxena (2017) found that the two home providers who experienced tension with their match (20%) did not feel comfortable communicating concerns directly with their home seeker or using mediation services, instead remaining in an uncomfortable living situation. Unwillingness to communicate appeared to maintain an unsatisfactory match (Bodkin & Saxena, 2017), and open communication-enabled match satisfaction (Macmillan et al., 2018).

Although studies spoke to the importance of open communication as enabling positive homeshare experiences, and lack of communication as posing a challenge, preferences with respect to best practices for effective communication differed between those who favored a more formalized approach involving matched homesharers jointly documenting and discussing household responsibilities, and those who preferred a more improvised approach, addressing behavior needing modification as required (Macmillan et al., 2018) or developing a routine over time involving shared activities and housekeeping without any schedule or written description of tasks for completion (Labit & Dubost, 2016).

### Intergenerational Engagement as Social Exchange

Three of the selected studies spoke to issues specific to intergenerational engagement (Labit & Dubost, 2016; Macmillan et al., 2018; Sánchez et al., 2011) and two were intergenerational in their design (Labit & Dubost, 2016; Sánchez et al., 2011). Intergenerational homesharing enabled the practice of intergenerational solidarity as measured along associational, affectual, and functional dimensions (Sánchez et al., 2011), resulting in a marked increase in frequency of intergenerational contact for home providers: home providers had contact with the young students with whom they cohabitated 13 times more frequently than with relatives under the age of 35, and 11 times more frequently than with nonfamilial individuals under the age of 35 (Sánchez et al., 2011). Results with respect to affectual solidarity were mixed: whereas 75% of students viewed older people more positively as a result of homesharing, 62% of home providers did not feel that living with a student improved their relationships with young people (Sánchez et al., 2011). However, 44.6% of those home providers felt more likely to partake in activities with younger cohorts, and 45.3% believed that they need to have greater interactions with younger people to feel good (Sánchez et al., 2011).

Findings from five studies spoke to home seeker–home provider intergenerational reciprocity (Bodkin & Saxena, 2017; Labit & Dubost, 2016; Macmillan et al., 2018; Rekart & Trevelyan, 1990; Sánchez et al., 2011). Within specifically intergenerational studies, altruistic motivations were expressed by home providers with respect to the difficulty young people face in finding housing in large urban areas (Labit & Dubost, 2016), and intergenerational learning was cited as both motivation (Labit & Dubost, 2016) and positive impact (Macmillan et al., 2018). Whereas 93.2% of elder home providers reported having benefitted from participation in intergenerational homesharing, 94% felt that they had benefitted the student as well, with 49% naming this benefit as emotional support (Sánchez et al., 2011).

### The Key Role of Agency Facilitation

Three studies spoke to the important role of agency involvement broadly in terms of feeling reassured by knowing that agency support was available, with staff being easily reached if needed (Bodkin & Saxena, 2017; Labit & Dubost, 2016; Macmillan et al., 2018), particularly in terms of problem-solving and conflict resolution (Bodkin & Saxena, 2017; Labit & Dubost, 2016) and ongoing monitoring of match progress (Labit & Dubost, 2016; Macmillan et al., 2018). Engaging in shared activities was a factor enabling and maintaining positive match relationships (Labit & Dubost, 2016; Macmillan et al., 2018; Rekart & Trevelyan, 1990), and home providers valued agency staff organizing events enabling socialization for matches (Labit & Dubost, 2016; Macmillan et al., 2018). Home providers reported choosing third-party arranged homesharing (TPAHS) over renting directly to tenants due to a sense of trust in both the TPAHS organization and the support services provided by staff, including vetting and matching services as well as availability of ongoing support.

Use of, and satisfaction with, match mediation differed among home providers: 6 of 10 home providers reported not needing match mediation (Bodkin & Saxena, 2017). Of the remaining four, two home providers used match mediation and positively described their experience and satisfaction with the mediation process and outcome as facilitated by agency staff, which included termination and re-matching for one home provider (Bodkin & Saxena, 2017). Respondents in research by Labit and Dubost (2016) also expressed positive feelings with respect to agency-facilitated conflict resolution. Home providers who reported feeling uncomfortable with their match did not use mediation or termination services, instead trying to manage the match relationship themselves despite reported discomfort with their home seekers (Bodkin & Saxena, 2017). See Table 2 for a further detailed thematic analysis of sources selected for inclusion.

**Table 2.** Thematic Analysis of Sources Selected for Inclusion

Source	Themes							
	Benefits of homeshare participation associated with			Challenges of homeshare participation associated with			Intergenerational engagement as social exchange	The key role of agency facilitation
	Companionship	Receiving support in daily tasks	Supplemental income	Sharing space	Sharing time	Interpersonal		
Altus and Mathews (2000)	X		X					
Bodkin and Saxena (2017)			X	X	X	X	X	X
Labit and Dubost (2016)		X		X			X	X
Macmillan and colleagues (2018)	X	X	X	X	X	X	X	X
Rekart and Trevelyan (1990)	X	X	X	X			X	
Sánchez and colleagues (2011)		X					X	

## Discussion

The objective of this scoping review was to identify the literature related specifically to the impacts of homesharing on older adults living in their homes. The six studies (four peer-reviewed, two gray literature) identified in this review demonstrated that the scope of the literature regarding homesharing is limited. This scoping review revealed four major themes (Table 2): (i) benefits of homeshare participation for older adults; (ii) challenges of participating in homeshare for older adults; (iii) intergenerational engagement as social exchange; and (iv) the key role of agency facilitation. Within these four themes, findings indicated that benefits of homeshare could be categorized as those associated with companionship, support in daily tasks, and supplemental income; the challenges of homesharing could be understood as one of navigating boundaries, in terms of sharing space and time, and navigating interpersonal boundaries in terms of lack of familiarity and difficulties with communication.

### Policy Implications

Results from this review spoke to the concept of aging in place, with results specifically demonstrating enhanced independence (Labit & Dubost, 2016; Macmillan et al., 2018; Rekart & Trevelyan, 1990; Sánchez et al., 2011). In particular, results from Sánchez and colleagues (2011) suggested that older adults engaging in homeshare had greater functional capacity in terms of being able to complete independent tasks of daily living when compared with a sample of the general population comparable in age and marital status. Operating on the supposition that the ability to age in place is contingent on having access to the social supports and services necessary to live safely and independently in the home (Social Development Canada, 2016) and that limited capacity to remain in the home may result in institutionalization, which is costly and overburdened (Rekart & Trevelyan, 1990), results indicating that the ability to continue living at home is enhanced by the services and supports provided within the homesharing exchange have implications for saving health and social services spending, as well as the potential for delaying and/or preventing premature institutionalization. HomeShare has been offered within the United States for more than 50 years, and Canadian nonprofit organizations have been offering homesharing services since 1980 (Gutman et al., 1989). Although there is currently a paucity of literature on homesharing user rates, research by Johnstone (2001) indicates that since 1980 there have been 35 HomeShare programs established in Canada, with 10 remaining in operation as of 2001. Given results indicating the potential for homeshare to enhance capacity to age in place, reduce risk of premature institutionalization for older adults, and save health and social service spending, results support policies that increase funding of—and access to—homesharing programs.

Findings specific to intergenerational homeshare (Labit & Dubost, 2016; Sánchez et al., 2011) are consistent with research by Jaffe (1989) in suggesting that intergenerational homeshare can effectively address the housing crisis of both property-rich, cash-poor older adults and less established younger adults, such as students, by providing both with safe, affordable housing without having to invest in any new developments or infrastructure, capitalizing instead on existing housing stock. Additionally, although traditional definitions of homesharing involve the home provider owning the home (Jaffe & Howe, 1988; McConnell & Usher, 1980), homesharing can also occur within shared rental agreements (Kaufman, 1983). Therefore, policies could be put in place to govern homeshare agreements that take place within the context of rented spaces, therefore expanding the potential scope of homesharing initiatives.

### Practice Implications

Ethical codes guiding most health practitioners mandate supporting an individual’s right to autonomy. The literature is definitive: older adults want to live at home for as long as possible (Bayer & Harper, 2000; CMHC, 2008) and, according to the Health Council of Canada (2012), they want to receive their care there. Therefore, implications of this review include serving as an evidence base to guide practitioner interventions and advocacy work supporting homeshare initiatives as a means of both upholding the autonomy of older adults and addressing pressing issues, such as the risk for social isolation. In addition, the specific needs of the most vulnerable (e.g., those with developmental disabilities, cognitive impairments, risk for isolation), can be prioritized in the matching process of a homeshare program, as these challenges are hard to address in the context of traditional housing arrangements. Furthermore, partnering with other service agencies across sectors (public, private, nongovernmental organizations) to oversee the matching of those who wish to participate in homesharing programs will allow for programs to continually address vulnerability, risk, and accountability, and to prioritize the needs of those who may be marginalized. Cross-sector collaboration also allows for complementary resources and perspectives to be meshed to address the need for technological innovation, and for stakeholders to remain responsive to the dynamic needs of specific (e.g., rural) programs. Results from this review speak to the facilitated model as key to supporting positive homesharing outcomes for this population, particularly by supporting match coherence through socialization and mitigating challenges associated with homesharing, particularly in terms of facilitating communication (Bodkin & Saxena, 2017; Labit & Dubost, 2016; Macmillan et al., 2018).

Findings indicating that home providers felt awkward communicating when wanting to end a match or needing to enforce exchange agreements and rules of conduct speak to the importance of the facilitated model as potential risk mitigation, because the inability to enforce rules and exchange agreements is rife with potential for abuse for a vulnerable older adult population. Furthermore, as optimal communication and preferred levels of formality with respect to documenting and enforcing exchange agreements varied among home providers (Labit & Dubost, 2016; Macmillan et al., 2018), dedicated agency support staff should tailor these processes to the individuals involved as much as possible. Research by Bodkin and Saxena (2017) suggests that further work into best practices for facilitating communication and ensuring home provider comfort with seeking mediation support is needed to ensure home providers do not remain in uncomfortable matches, a circumstance with potential for abuse.

In a study that explored the factors contributing to problematic intergenerational homeshare matches, Jaffe and Wellin (1989) found that success or failure of a match was associated with whether home providers’ expectations for support and companionship were met or unmet by their home seekers. These findings by Jaffe and Wellin (1989), with respect to mismatched expectations in relation to companionship and support, were echoed in results within this scoping review (Bodkin & Saxena, 2017; McConnell & Usher, 1980), suggesting that further work around expectation-management is needed by agency staff.

Finally, results by Bodkin and Saxena (2017) indicating difficulty experienced by older adult home providers due to lack of familiarity with what they perceive to be the home seeker’s mental health challenges or a home seeker’s status as a trans person suggest that navigating interpersonal and cultural differences poses a challenge within homeshare match relationships. More work needs to be done in ensuring careful prematch evaluation to ensure best match fit, and training or processes, such as prematch psychoeducation and cultural competency training, to ensure the comfort and safety of both home seekers and home providers.

### Research Implications

Due to the limited nature of the services home seekers are able to provide (or may agree to provide, without becoming caregivers), homesharing requires considerable independence from its participants (Gutman et al., 1989). Findings of this scoping review suggested that home providers with impairment, either preexisting or developing throughout the duration of the match, had needs exceeding the level of support that can be provided by a home seeker within the context of the homeshare programs in this review. Research exploring differences in exchange arrangements and satisfaction between “traditional” arrangements (between the home provider and lodger) and “caregiving” arrangements (usually between older, more frail older adults and involving caregiving) as interpreted through social exchange theory (Danigelis & Fengler, 1990, 1991) may lend insight into the needs and experiences of more dependent home providers in order to optimize programs to serve this population. Furthermore, findings from Altus and Mathews (2000) suggest that age of the home provider may matter in terms of needs and impact, and further study is needed to examine differences with respect to the experience of homesharing among different age cohorts of home providers. This is especially pressing given demographic shifts and an expected rise in the population aged 85 and older, often referred to as the oldest old: according to Statistics Canada (2017a), baby boomer cohorts (those born between 1946 and 1965) will reach age 85 starting in 2031 and by 2051, when the youngest baby boomers reach age 85, almost 2.7 million people, or 5.7% of the population in Canada, will likely be aged 85 and older, signaling a need to provide services and supports geared toward addressing issues specific to older, potential more frail populations. Furthermore, findings of this scoping review were impacted by small sample sizes and limited generalizability, necessitating more large-scale studies, and all findings in this review were based on self-reported outcomes. Altus and Mathews (2000) suggested the need for future research examining actual performance data, such as time spent socializing, leaving the home, or engaging in certain activities.

## Limitations

A broad research question as well as broad subquestions guided this scoping review, and findings were reviewed descriptively. Literature included English-language texts published between 1989 and December 2018. Furthermore, we limited our population of study to home providers aged 55 and older, therefore excluding results from program evaluations for homeshare programs that, while predominantly servicing an older adult population, do not have minimum age requirements for program participation. Additionally, studies from outside of Canada were used to inform research, practice, and policy implications as understood through a Canadian lens. The generalizability and applicability of these studies to a Canadian context or to that of another jurisdiction may be limited by differences in policy and practice, as well as cultural differences across regions. However, due to the limited literature on the questions framing this scoping review, an examination of all relevant research was necessary, with findings from studies originating outside of Canada serving to signal areas for further inquiry. Lastly, despite efforts toward conducting a comprehensive scan of both peer-reviewed and gray literature, it is possible that not all relevant sources were located for inclusion.

## Conclusion

The purpose of this review was to describe and synthesize the existing literature in this area and to identify current best practices and interventions. Results indicated that older adults benefitted from the companionship, supplemental income, and support received through homeshare, that navigating boundaries with respect to sharing space, time, and interpersonal relationships was a challenge when homesharing, and that agency facilitation was key to supporting a positive homeshare experience for older adults. Taken together, findings were used to derive implications for policy, practice, and to highlight areas for future research, indicating that homesharing has implications for saving health and social services spending, that further work into best practices to facilitate communication and ensure home provider comfort with seeking mediation support is needed, and that further research into the experience of homesharing for older, more impaired older adults is required. By shifting the discussion from program-oriented outcomes to person-oriented impacts, centering focus onto the older adults themselves and the ways homesharing impacts their lives and capacity to age in place, we are able to better determine the viability of homeshare as a potential solution for improving and prolonging for older adults the experience of living in their own home.

## Supplementary Material

igaa011_suppl_Supplementary_Material
