# Supplementary material for: More Than Just a Room: A Scoping Review of the Impact of Homesharing for Older Adults
Source: Innov Aging. 2020 May 3;4(2):igaa011. doi: 10.1093/geroni/igaa011 (PMC7196182; doi:10.1093/geroni/igaa011)
Supplement: igaa011_suppl_Supplementary_Material [file igaa011_suppl_supplementary_material.doc]

**Supplementary Material**

| **Appendix A. Extraction table of characteristics of final selected sources** | | | | | |
| --- | --- | --- | --- | --- | --- |
| Citation | Country of Origin | Source Type | Study Objective | Study Details | Key Findings |
| Altus, D.E., & Mathews, R.M. (2000). Examining satisfaction of older home owners with intergenerational homesharing. *Journal of Clinical Geropsychology*, *6*(2), 139-147. | United States | Cross-sectional study (survey) | 1. To examine if, as a result of their experience homesharing, older adult homeowners would report changes across five domains of life satisfaction: well-being, health, engagement in activities of daily living, social activities, and financial.  2. To determine whether satisfaction dimensions would vary with respect to age, gender, and match length. | A survey was mailed to the entire pool of homeowners currently engaged in active homesharing matches through three homesharing programs (N = 252). The response rate was 42 percent. Only surveys from respondents reporting their age as 50 and over (*n* = 93) were included for analysis.  The 21-item survey asked respondents their age, gender, length of current homesharing match, as well as to rate whether they experienced positive, negative, or no change with respect to the five life satisfaction domains as a result of homesharing.  Overall results were further analyzed by age, gender, and length of current homesharing match. | Results from the overall study sample did not meet our age-specific inclusion criteria and were therefore outside the scope of this review.  Results stratified by age revealed a statistically significant difference between responses from participants aged 50 to 69 (*n* = 41, 44%) compared to those from participants aged 70 and over (*n* = 52, 56%).  Older respondents were significantly more likely to report feeling safer, liking living in their home more, eating better, following the news more, and watching more television as a result of their homesharing experience. Younger respondents were significantly more likely to report worrying less about finances, being better off financially, and spending more time doing housework. |
| Bodkin, H. & Saxena, P. (2017). Exploring home sharing for elders. *Journal of Housing for the Elderly*, *31*(1), 47-56 | United States | Qualitative research design (open-ended interview) | 1. To examine the experience of third-party arranged homesharing (TPAHS) for older adult home providers sharing their homes with home seekers of any age.  2. To provide recommendations to enhance the TPAHS experience for this population. | Participants (*n*=10) were homeowners 65 years of age and older currently participating (*n* = 8, 80%), or who had participated (*n* = 2, 20%) in a TPAHS program as home providers within a year prior to the study start date.  Data was collected via a 60-90 minute 17-question semi-structured interview containing open-ended questions regarding the participants’ TPAHS experience and follow-up questions adapted to the content of each participant’s initial responses. Qualitative data analysis of responses was conducted. | 1. Motivating factors for taking part in TPAHS included a desire for: improving the experience of living at home, companionship, help with tasks of living independently, increased sense of security, financial incentive, and a relatively inexpensive way to receive assistance compared to the cost of hiring professional care.  2. The majority of participants reported positive feelings toward their home seeker.  3. Six home providers (60%) reported not needing match mediation. Out of four participants involved in self-reported unsatisfactory matches, two sought match mediation by TPAHS staff and positively described their experience. The remaining two did not use mediation and remained in the match despite discomfort with their home seeker.  4. Three participants reported hosting home seekers with what they perceived as mental health challenges. Each home provider experienced this differently, depending on their own experience with mental health challenges and their level of comfort seeking match support.  5. The two participants who were blind reported experiencing loneliness, boredom, and increased need for human interaction. One blind home provider hired his former TPAHS home seeker as a live-in companion in order to meet his care and companionship needs exceeding that which could be provided under TPAHS. |
| Labit, A., & Dubost, N. (2016). Housing and ageing in France and Germany: the intergenerational solution. *Housing, Care and Support*, *19*(2), 45-54. | France | Qualitative research design (Field survey with semi-structured interview) | To explore the reasons for the differences between how intergenerational housing is being implemented in France and Germany and to present results from field surveys conducted in each of these countries. | The German intergenerational housing scheme did not meet our inclusion criteria.  In France, a qualitative field survey consisting of semi-structured interviews was undertaken in 2011-2012 of stakeholders from the intergenerational homesharing organization, "At-Home Crochus".  Two student-senior pairings were interviewed. The two older adults were women, aged 72 and 80. The two young persons were both 19, one male and one female. | 1. Both seniors emphasized safety and enhanced sense of security as benefits of homesharing. One older adult cited having someone to care for her pet when she travelled as a benefit.  2. Both pairings cited facilitation and agency involvement as key, particularly in terms of conflict resolution and in organizing social events for program participants to meet and socialize.  3. Reported motivating factors for homesharing for the older adults included wanting to interact with and learn from a young person and sympathy for the difficulties obtaining housing faced by contemporary young people. |
| Macmillan, T., Gallagher, J., Ronca, M., Bidey, T., & Rembiszewski, P. (2018). *Evaluation of the Homeshare pilots: Final report.* Shared Lives Plus. | United Kingdom | Report/Program Evaluation (mixed methods research design using primary qualitative research and secondary analysis of data sets) | To determine factors contributing to a sustainable homesharing program by (1) identifying the value of homesharing; (2) identifying best practices for homesharing. | 12 homeshare matches and two individuals from separate matches (*n*= 26) from UK Oxfordshire, PossAbilities, and Novus, were interviewed at baseline and endline phases regarding their homeshare experience.  The average age of home providers was 81. The average age of home seekers was 34. There was some variation in terms of homesharers being interviewed: of all interviews conducted, eight home providers and seven home seekers were interviewed at both baseline and endline phases. | 1. Home providers reported benefits of homesharing as improved wellbeing (better moods, reduced anxiety, improved sleep, and increased activity and confidence in mobility), increased companionship and reduced loneliness, support given with physical tasks around the house, and intergenerational learning (support with technology).  2. Agency support, maintaining personal space, open intra-match communication, and engaging in activities together were match enablers.  3. Difficulties with sharing space, increased needs of home providers during the life of a match, timing of support, and awkwardness directly communicating concerns were found to be challenges of homesharing. |
| Rekart, J., & Trevelyan, S. (1990). *Homesharing and quality of life; the perspective of seniors*. Vancouver, Canada; Social Planning and Research Council of British Columbia. | Canada | Report (Survey) | 1. To describe services provided by the Vancouver Homesharers Society, provide demographic information on its clients, examine client perceptions of benefits and challenges of homesharing, and to compare findings with data collected from other Canadian and US studies on homeshare.  2. To gather baseline data on quality of life (QoL) of matched home providers to be used in a future study in order to measure the impact of homesharing on QoL. However, a future study was not conducted. | Study participants were randomly selected from clients of the Vancouver Homesharers Society, producing a sample of matched home providers aged 55 and over (*n*=37) and matched home seekers (*n*=27) and a control group of 15 unmatched home providers on the waiting list (all 55+). 75 percent of matches were intergenerational.  Participants completed a one-hour interview asking participant personal characteristics, perceptions of homesharing, and health status or QoL. Open-ended questions were used to solicit qualitative data on the advantages and disadvantages of homesharing for matched homesharers. | Benefits reported by home providers: the positive feelings associated with increased companionship and reduced social isolation (mentioned by 59.5%), assistance with household tasks (35.1%), security (29.7%), and enhanced independence (22.6%).  Main reported disadvantage of homesharing by home providers: loss of privacy or loss of control over the home (mentioned by 32.4%). 51.4 percent of home providers denied experiencing any drawbacks to homesharing.  The majority of matched home providers were satisfied with homesharing (75%) and expressed satisfaction in terms of feeling safer in their home (75%), companionship (61%), reduced loneliness (61%), the desire to help the home seeker (60%), and satisfaction with light housework completed by the home seeker (52%). |
| Sánchez, M., García, J.M., Díaz, P., & Duaigües, M. (2011). Much more than accommodation in exchange for company: dimensions of solidarity in an intergenerational homeshare program in Spain. *Journal of Intergenerational Relationships, 9*(4): 374-388*.* | Spain | Cross-sectional study (Survey research design consisting of open-ended and close-ended questionnaire administered by interview) | To examine data from the evaluation of Viure i Conviure ("Live and Live Together" [ViC]), Spain's largest intergenerational home share program, and assess whether intergenerational cohabitation, as experienced by ViC participants, enables the practice of intergenerational solidarity as measured along associational, affectual, and functional dimensions. | A sample (*n*=306, 149 home providers and 157 home seekers) was randomly selected from current ViC homesharers (N=658). From the sample, home providers were aged 56-99 (mean age = 82.2). Home seekers were aged 18-44 (mean age = 25.0).  The study used structured interviews based on two questionnaires consisting of both open- and close-ended questions relating to study indicators of intergenerational solidarity as measured along associational, affectual, and functional dimensions.  Two indicators were created for each dimension of solidarity to describe its practice, each indicator was measured from the perspective of each member of the matched pair: Associational solidarity was measured by reported frequency of intergenerational contact; Affectual solidarity was indicated by perceived improvement in intergenerational personal relationships; Functional solidarity was measured by the percentage of home providers escorted to places and receiving assistance by their matched home seeker.  Interview data was gathered and responses from open-ended questions were coded and analyzed using statistical processing. | 1.ViC participation was found to enable increased frequency of intergenerational contact, therefore resulting in enabling associational intergenerational solidarity.  2. Affectual intergenerational solidarity: In terms of participants' perception of improvements in their intergenerational relationships, results were mixed. 47.3% of older adults and 75.8% of students report seeing the other group (young people and older adults, respectively) more positively. 62.2% of home providers did not believe that sharing their home with a student resulted in improved relationships with young people as compared to before participating in ViC.  3. Functional intergenerational solidarity was enabled by ViC participation for both the older adults and students. Both groups reported providing support to the other, either emotionally or practically. Compared to a sample Spanish population of comparable age and marital status, older ViC participants reported greater capacity of completing tasks of daily living. Findings suggest that increased capacity was somehow associated with service provision by the ViC students and that the older participants experienced increased capacity to carry out daily tasks of independent living when able to depend on access to appropriate assistance. Further, 93.2% of elders reported that participating in homeshare had benefitted them. Of these respondents, 51.7% reported the greatest benefit as help received with personal care activities. |

**Appendix B**

Searches and Search Strings as Adapted in Accordance with Search Parameters of each Database

**EBSCO-Hosted Databases**

**AgeLine.** The following searches were conducted by search (S) number:

S9. S4 AND S8

S8. S5 OR S6 OR S7

S7. AB ((older N1 (adult* OR people OR person)) OR senior* OR elder* OR aging OR geriatric OR gerontolog*))

S6. TI ((older N1 (adult* OR people OR person)) OR senior* OR elder* OR aging OR geriatric OR gerontolog*))

S5. (DE "Older Adults")

S4. S1 OR S2 OR S3

S3. AB (((home* N1 shar*) or homeshar* or sharehome) or (flat N1 shar*) or ((share* N1 hous*) or houseshar*) or (communal N1 (hous* or living)) or (roommate* or (room N1 mate*)) or (housemate* or (house N1 mate*)) or (cohousing or (co N1 housing)))

S2. TI (((home* N1 shar*) or homeshar* or sharehome) or (flat N1 shar*) or ((share* N1 hous*) or houseshar*) or (communal N1 (hous* or living)) or (roommate* or (room N1 mate*)) or (housemate* or (house N1 mate*)) or (cohousing or (co N1 housing)))

S1. (DE "Shared housing")

**CINAHL Plus with Full Text.** The following searches were conducted by search (S) number:

S8. S3 AND S7

S7. S4 OR S5 OR S6

S6. AB ((older N1 (adult* OR people OR person)) OR senior* OR elder* OR aging OR geriatric OR gerontolog*))

S5. TI ((older N1 (adult* OR people OR person)) OR senior* OR elder* OR aging OR geriatric OR gerontolog*))

S4. ((MH "Aged") OR (MH "Aged, 80 and Over+") OR (MH "Middle Age"))

S3. S1 OR S2

S2. AB (((home* N1 shar*) or homeshar* or sharehome) or (flat N1 shar*) or ((share* N1 hous*) or houseshar*) or (communal N1 (hous* or living)) or (roommate* or (room N1 mate*)) or (housemate* or (house N1 mate*)) or (cohousing or (co N1 housing)))

S1. TI (((home* N1 shar*) or homeshar* or sharehome) or (flat N1 shar*) or ((share* N1 hous*) or houseshar*) or (communal N1 (hous* or living)) or (roommate* or (room N1 mate*)) or (housemate* or (house N1 mate*)) or (cohousing or (co N1 housing)))

**OVID-Hosted Databases**

**MEDLINE: Epub Ahead of Print, In-Process & Other Non-Indexed Citations, MEDLINE® Daily and MEDLINE® 1946-Present.** The following searches were conducted (by search number):

1. ((home$1 adj1 shar$3) or homeshar* or sharehome or (flat adj1 shar$3) or ((share$3 adj1 hous$3) or houseshar$3) or (communal adj1 (hous$3 or living)) or (roommate$1 or (room adj1 mate$1)) or (housemate$1 or (house adj1 mate$1)) or (cohousing or (co adj1 housing))).tw,kf.

2. exp AGED/ or exp Middle Aged/ or exp "AGED, 80 AND OVER"/

3. ((older adj1 (adult* or people or person*)) or senior* or elder* or aging or geriatric or gerontolog*).tw,kf.

4. 2 or 3

5. 1 and 4

**Social Work Abstracts (1968 to December 2018).** The following searches were conducted (by search number):

1. ((home$1 adj1 shar$3) or homeshar* or sharehome or (flat adj1 shar$3) or ((share$3 adj1 hous$3) or houseshar$3) or (communal adj1 (hous$3 or living)) or (roommate$1 or (room adj1 mate$1)) or (housemate$1 or (house adj1 mate$1)) or (cohousing or (co adj1 housing))).tw,sh.

2. ((older adj1 (adult* or people or person*)) or senior* or elder* or aging or geriatric or gerontolog*).tw,sh.

3. 1 and 2

**ProQuest-Hosted Databases**

**Applied Social Sciences Index and Abstracts (ASSIA) (1987 – current), Dissertations & Theses @ University of Toronto‎; ERIC (1966 - current), Policy File Index (1990 - current), ProQuest Dissertations and Theses Global; PsycINFO (1806 - current); Social Services Abstracts (1979 - current), Sociological Abstracts (1952 – current**). The following search string was employed:

TI,AB(((home* NEAR/1 shar*) OR homeshar* OR sharehome) OR (flat NEAR/1 shar*) OR ((share* NEAR/1 hous*) OR houseshar*) OR (communal NEAR/1 (hous* OR living)) OR (roommate* OR (room NEAR/1 mate*)) OR (housemate* OR (house NEAR/1 mate*)) OR (cohousing OR "co housing")) AND TI,AB((older NEAR/1 (adult* OR people OR person) OR senior* OR elder* OR aging OR geriatric OR gerontolog*))

**Additional Grey Literature Databases**

**Google Advanced Scholar.** Separate searches were conducted using the search strings listed below. The first page of results for the shorter searches and the first 100 results for the longer search string below were initially reviewed for relevance to the question of our scoping review and adherence to our inclusion and exclusion criteria.

- Older adult AND homeshar*
- Older adult AND cohous*
- Senior AND homeshar*
- Senior AND cohous*
- Elderly AND homeshar*
- Elderly AND cohous*
- (home* shar* OR shar* home* OR homeshar* OR home-shar* OR sharehome OR flat shar* OR shar* flat OR flat-shar* OR share* hous* OR hous* shar* OR house-shar* OR houseshar* OR communal hous* OR communal living OR roommate* OR room mate* OR room-mate* OR housemate* OR house mate* OR house-mate* OR cohousing OR co-housing) AND (older adult* OR senior* OR elder* OR aging OR older people OR older person OR geriatric OR gerontolog*)

**OpenGrey Repository (Europe)**. The following search string was employed:

(((home* NEAR/1 shar*) OR homeshar* OR sharehome) OR (flat NEAR/1 shar*) OR ((share* NEAR/1 hous*) OR houseshar*) OR (communal NEAR/1 (hous* OR living)) OR (roommate*) OR (room NEAR/1 mate*)) OR (housemate* OR (house NEAR/1 mate)) OR (cohousing OR "co-housing")) AND ((old* NEAR/1 adult* OR people OR person) OR senior* OR elder* OR aging OR geriatric OR gerontolog*))

**Scopus.** The following search string was employed:

(TITLE-ABS-KEY ("home* W/1 shar*" OR homeshar* OR sharehome OR "flat W/1 shar*" OR "share* W/1 hous*" OR houseshar* OR ("communal W/1 (hous* OR living)") OR (roommate* OR "room W/1 mate*" OR housemate* OR "house W/1 mate" OR cohousing OR "co-housing"))) AND (TITLE-ABS-KEY (("older W/1 (adult* OR people OR person)" OR senior
